# Seronegative antibody‐mediated neurology after immune checkpoint inhibitors

**DOI:** 10.1002/acn3.547

**Published:** 2018-03-25

**Authors:** Robert Wilson, David A. Menassa, Alexander J. Davies, Sophia Michael, Joanna Hester, Wilhelm Kuker, Graham P. Collins, Judith Cossins, David Beeson, Neil Steven, Paul Maddison, Simon Rinaldi, Saiju Jacob, Sarosh R. Irani

**Affiliations:** ^1^ Oxford Autoimmune Neurology Group Nuffield Department of Clinical Neurosciences University of Oxford John Radcliffe Hospital Oxford United Kingdom; ^2^ Nuffield Department of Surgical Sciences University of Oxford John Radcliffe Hospital Oxford United Kingdom; ^3^ Neuroradiology Department Oxford University Hospitals Oxford United Kingdom; ^4^ Department of Clinical Haematology Churchill Hospital Oxford United Kingdom; ^5^ Department of Clinical Oncology University Hospitals Birmingham Birmingham United Kingdom; ^6^ Department of Neurology Queen's Medical Centre Nottingham United Kingdom; ^7^ Centre for Rare Diseases and Queen Elizabeth Neuroscience Centre University Hospitals Birmingham Birmingham United Kingdom

## Abstract

Checkpoint inhibitor medications have revolutionized oncology practice, but frequently induce immune‐related adverse events. During autoimmune neurology practice over 20 months, we prospectively identified four patients with likely antibody‐mediated neurological diseases after checkpoint inhibitors: longitudinally extensive transverse myelitis, Guillain–Barré syndrome, and myasthenia gravis. All patients shared three characteristics: symptoms commenced 4 weeks after drug administration, responses to conventional immunotherapies were excellent, and autoantibodies traditionally associated with their syndrome were absent. However, serum immunoglobulins from the myelitis and Guillain–Barré syndrome patients showed novel patterns of tissue reactivity. Vigilance is required for antibody‐mediated neurology after checkpoint inhibitor administration. This phenomenon may inform the immunobiology of antibody‐mediated diseases.

## Introduction

A major recent advance in oncology has been the success of immune checkpoint inhibitors. These drugs are increasingly used to treat various cancers including melanoma, kidney adenocarcinoma, lung carcinomas, and hematological malignancies.^1^ They commonly target programmed death 1 (PD‐1) or cytotoxic T‐lymphocyte antigen‐4 (CTLA‐4) and, more rarely, PD‐ligand 1 (PD‐L1). PD‐1, and CTLA‐4 are expressed in both conventional T cells and regulatory T cells (Tregs), among other cell types.^2^, ^3^ However, immune‐related adverse events (irAEs) affect up to 40% of patients treated with checkpoint inhibitors and include colitis, dermatitis, pneumonitis, and hepatitis.^4^ More rarely, neurological side effects are observed: the most frequent is hypophysitis.^5^ While conventional models typically suggest irAEs are T cell mediated, here, we describe likely antibody‐mediated autoimmunity with the first case of immune checkpoint‐blockade associated with longitudinally extensive transverse myelitis (LETM, *n* = 1), and cases who developed myasthenia gravis (MG, *n* = 2) and Guillain–Barré syndrome (GBS, n = 1) after checkpoint inhibitors. The escalating use of these drugs in oncology requires heightened vigilance among neurologists for these associated, often seronegative, autoantibody‐mediated side effects that respond well to conventional immunotherapies.

## Patients and Methods

Four patients prospectively observed during routine Autoimmune Neurology practice between July 2015 and March 2017 administered immune checkpoint inhibitors are summarized in Table 1. We performed blinded testing of serum for autoantibodies in all patients, almost exclusively using previously described live cell‐based assay methodologies (Table 1),^6^, ^7^ and flow cytometry from whole blood in patient 1. Written informed consent was obtained with ethical approval (REC16/YH/0013).

**Table 1:** Clinical and investigation features of patients with neurological complications after checkpoint inhibitors

	Patient 1	Patient 2	Patient 3	Patient 4
Age	35	57	62	52
Sex	Male	Male	Female	Male
Tumor	Classical Hodgkin lymphoma	Melanoma	Lung adenocarcinoma	Melanoma
Checkpoint inhibitor(s)	Pembrolizumab	Nivolumab and ipilimumab	Pembrolizumab	Nivolumab and ipilimumab
Time from administration to symptoms	4 weeks	4 weeks	4 weeks	4 weeks
Clinical features	Tetraparesis, sensory level, loss of sphincters	Fatigable ptosis and complex external ophthalmoplegia	Fatigable ptosis and limb weakness	Sensory loss and reduced reflexes
Clinical diagnosis	Longitudinally extensive transverse myelitis	Myasthenia gravis	Myasthenia gravis	Guillain–Barre syndrome
Novel serum autoantibody?	Yes, IgG	No	No	Yes, IgM
Negative antibody results	AQP4, MOG, CRMP5, GFAP, amphiphysin	AChR (including clustered), MuSK, LRP4	AChR (including clustered), MuSK, LRP4	Gangliosides, CRMP5, GFAP, Contactin‐1, CASPR1, NF140/155/186
Nerve conduction/EMG studies	Not performed	Normal	Normal	Prolonged distal motor latencies, low conduction velocities and absent F waves.
Treatment for neurological features	Corticosteroids, intravenous immunoglobulins and plasma exchange	Corticosteroids	Pyridostigmine and corticosteroids	Intravenous immunoglobulins and corticosteroids
Oncological Outcome	Complete remission	Death, progression of metastases	Stable lung tumor, resolution of metastases	Reduction in tumor load
Neurological Outcome	Excellent, mild residual hypertonia	Complete	Complete	Complete
Follow‐up period	2.5 years	6 months	1 year	1 year

Patient 1 received adriamycin, bleomycin, vinblastine, dacarbazine, then ifosfamide, epirubicin, and etoposide, and finally, brentuximab (CD30 targeting). No relapses of the tumor or neurology were noted. Aquaporin 4 (AQP4) antibodies were not detected on live and fixed cell‐based assays. CRMP5 and amphiphysin antibodies were tested by commercial line blot, and GFAP antibodies by fixed cell‐based assay. Other antibodies were tested by live cell‐based assays.^6^, ^7^ Patient 3 had no single fiber EMG performed, and EMG studies in both patients 2 and 3 were performed after treatment initiation. AChR, acetylcholine receptor; CASPR1, contactin‐associated protein 1; CRMP5, collapsin response mediator protein 5; GFAP, glial fibrillary acidic protein; LRP4, low‐density lipoprotein receptor‐related protein 4; MOG, myelin oligodendrocyte glycoprotein; MuSK, muscle‐specific kinase; NF, neurofascin.

## Results

### Clinical features

**Figure 1:**
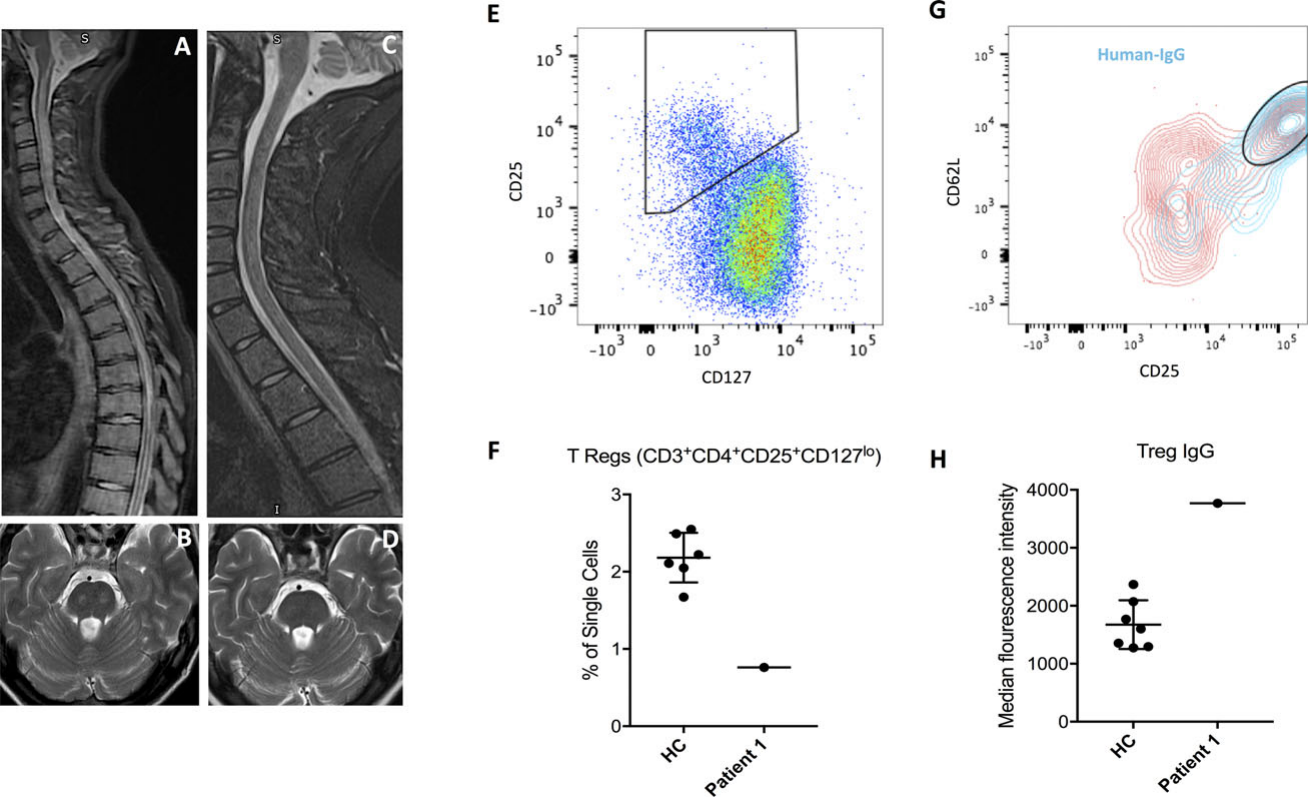
Radiological and immunological features of patient 1**.** (A) T2‐weighted STIR sequence with extensive medullary cord edema and swelling extending from the medulla oblongata into the low thoracic spinal cord. (B) The pons also shows T2‐weighted bilateral circumscribed areas of high signal on axial imaging. (C and D) After 4 months and corticosteroids, intravenous immunoglobulins and plasma exchange, both sagittal and axial images show almost complete resolution of swelling with no persistent gliosis or atrophy. (E) Flow cytometry plots show CD3^+^ CD4^+^ T cells gated on CD25 and CD127 to identify Tregs (CD25^+^ CD127^lo^) in healthy control (HC) peripheral blood. (F) Enumeration revealed a reduction of Tregs in the blood of patient 1. (G) Tregs from patient 1 contained a subpopulation of CD25^++^ CD62L^++^ cells, all of which stained positively for human IgG, representing bound humanized pembrolizumab (blue contours). Red contours represent population which were not bound by human IgG. (H) This population was not bound by IgG in the healthy controls.

Patient 1 was a 35‐year‐old male diagnosed in 2005 with stage IIa Hodgkin lymphoma which relapsed despite three lines of chemotherapy over 10 years (Table 1). Residual metastases prompted initiation of pembrolizumab (a humanized PD‐1 monoclonal antibody) as fourth line therapy, with two cycles given at 3 week intervals. One week after the second cycle, he developed acute urinary retention, constipation, hiccoughs, and vomiting, with weakness and sensory loss in arms and legs. Examination revealed a spastic tetraparesis with profound sensory loss and sphincter atonia. MRI showed a LETM from the pons to the lower thoracic spine with extensive cord edema (Fig. 1A–B). Aquaporin‐4 and myelinoligodendrocyte glycoprotein antibodies were not detected. CSF showed 24 mononuclear cells/mm^3^; other detailed CSF and blood tests were unremarkable (Table S1). Intravenous methylprednisolone and, subsequently, plasma exchange were administered with the aim of depleting free circulating pembrolizumab and any putative autoantibody. During an oral prednisolone taper, repeat MRI at 6 months showed considerable reduction of edema (Fig. 1C–D). One year from symptom onset, he was continent, independent in ambulation, and his Hodgkin lymphoma had responded to the treatment.

To explore the underlying immunology, flow cytometry was performed from the patient's peripheral blood mononuclear cells at the nadir of the neurological syndrome. This revealed a decrease in Treg numbers (CD3^+^CD4^+^CD25^+^CD127^lo^; Fig. 1E–F) and an anti‐human‐IgG antibody preferentially bound to the residual Tregs that expressed the highest levels of CD62L and CD25, indicating a subpopulation targeted by the humanized pembrolizumab (Fig. 1G–H).

Patient 2 was a 57‐year‐old male with metastatic melanoma involving the left axilla, mediastinum, and lung hilum. Four weeks after the first infusion of nivolumab (a humanized PD‐1 monoclonal antibody; at 1 mg/kg) and ipilimumab (a CTLA‐4 blocking antibody; at 3 mg/kg), and 1 week after the second course, he developed exertional breathlessness with diplopia and ptosis which worsened toward the end of the day. Examination showed weak left eye abduction, right eye adduction, and bilateral asymmetrical fatigable ptosis. The remainder of the examination and investigations were unremarkable. A clinical diagnosis of MG prompted intravenous methylprednisolone followed by 80 mg oral prednisolone, with a rapid improvement in all features. All known MG‐associated autoantibodies were negative (Table 1). The corticosteroids were gradually tapered. Patient 3 also developed seronegative MG, with limb weakness and ptosis, 4 weeks after the first dose of pembrolizumab for metastatic lung adenocarcinoma. Complete remission was achieved 3 months after commencing pyridostigmine and corticosteroids.

Patient 4 was a 52‐year‐old male with metastatic melanoma affecting the ear, cervical lymph nodes, and lung. Treatments included radical neck dissection, and nivolumab with ipilimumab. Three weeks after the first infusion, and 2 days after the second cycle, he developed headache and generalized tiredness. Investigations revealed hypophysitis and prednisolone was commenced. A week later, he experienced progressive hand and feet numbness, with bilateral facial weakness, distal limb weakness, and reduced deep tendon reflexes. CSF was acellular with a protein content of 2.3 g/L. Neurophysiology revealed a demyelinating neuropathy and both whole spine MRI and ganglioside antibodies were unremarkable (Table 1). In summary, the findings were consistent with the acute inflammatory demyelinating polyneuropathy variant of GBS. He was commenced on intravenous immunoglobulins with a very good recovery.

In all four patients, the checkpoint inhibitors were discontinued and no neurological relapses were noted at follow‐up (0.5–2.5 years, Table 1).

These 4 cases were observed over a 20 month period, and this exceeded the cumulative frequency of patients with antibodies against dipeptidyl‐peptidase‐like protein‐6 (DPPX, *n* = 1), the *α*‐amino‐3‐hydroxy‐5‐methyl‐4‐isoxazolepropionic acid receptor (AMPAR, *n* = 0), the gamma‐aminobutyric acid A‐receptor (GABA_A_R, *n* = 1), and Iglon5 (*n* = 0).^8^

### Novel autoantibody detection

**Figure 2:**
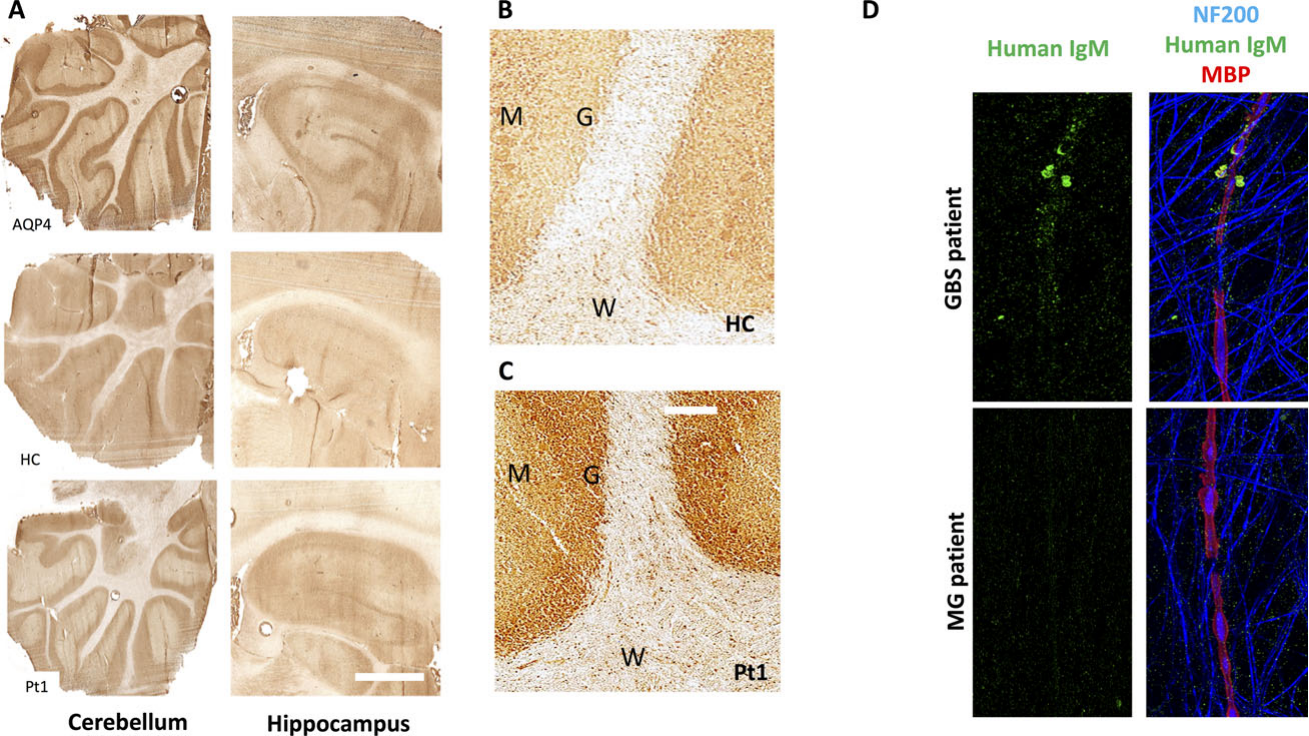
Novel autoantibody reactivities in patients with checkpoint inhibitors. (A) Aquaporin‐4 antibody positive (AQP4), healthy control (HC), and patient number 1 (Pt1) serum IgG binding to rodent sections of cerebellum (left column) and hippocampus (right column). Scale bar = 500 *μ*m. Paraformaldehyde‐fixed brain sections were incubated for 1 hour with patient serum (1:200 dilution in PBS/0.1% Triton‐X100/5% bovine serum albumin), washed in PBS/0.1% Triton X100, and then incubated with anti‐human HRP‐conjugated secondary antibodies (1:750) for 45 min. Visualization with 3,3′‐diaminobenzidine and hydrogen peroxide. (B) Higher magnification showing HC and patient 1 (Pt1; C) IgG binding to cerebellar granule cells (G) more than molecular layer (M) or white matter (W). Scale bar = 100 *μ*m. (D) Serum IgM (1:100 dilution for 1 h at 37°C) from the patient with Guillain–Barré syndrome bound live myelinating cocultures (from human‐induced pluripotent stem cell‐derived sensory neurons and rat primary Schwann cells). Subsequently, cultures were fixed in 1% paraformaldehyde and labeled with AlexaFluor‐488 anti‐human conjugated antibodies (green). The observed binding was to myelin blebs in particular. This was followed by permeabilization with ice‐cold methanol (30 min, on ice), and counter‐labeling anti‐neurofilament‐heavy (1:10,000, labeled blue, NF200) and anti‐myelin basic protein (1:500, labeled red, MBP) primary antibodies to visualize axonal processes and myelin internodes, respectively.

Serum immunoglobulin G exclusively from the patient with LETM bound to rodent brain tissue in a pattern comparable to aquaporin‐4 (Fig. 2A–C). In addition, serum immunoglobulin M from the GBS patient, but not the other three patients, bound myelinating cocultured induced pluripotent stem cell‐derived sensory neurons and rat primary Schwann cells (Fig. 2D), prepared as previously described.^9^ These reactivities were not seen in 20 healthy controls. No patient immunoglobulins bound to rodent muscle sections, C2C12 myotubes or CN21 muscle cell lines.

## Discussion

Immune checkpoint inhibitors provide an increasingly popular and contemporary approach to effective treatment of many malignancies. This approach increases patient survival but carries risks of irAEs. These complications need to be actively recognized by neurologists in an era with increasing use of these medications in the routine clinical oncology setting, and are likely to be more common than many of the recently described antibody‐mediated illnesses.^8^

The patients we report showed a range of classical antibody‐mediated conditions of the central and peripheral nervous system, and shared three intriguing features. First, there was a stereotyped 4‐week lag from immune checkpoint inhibitor administration to symptom onset, which has mechanistic implications discussed below. Second, and by contrast to a recent series in MG,^10^ despite clinical presentations and investigation findings indistinguishable from traditional seropositive equivalents, none showed the common autoantibody profiles associated with their respective conditions. However, two patient sera showed novel autoantibody reactivities with disease‐relevant preparations, including one on a live cell system, suggesting study of these patients may hasten the discovery of antigenic targets in conventional antibody‐mediated diseases. Finally, all patients showed a very good recovery with symptomatic therapies or immunotherapies including corticosteroids, intravenous immunoglobulins, and/or plasma exchange, without residual clinical deficits or atrophy. This consistent improvement, in addition to the novel observed autoantibody reactivities, suggest antibody‐mediated effector neurological mechanisms rather than the more commonly hypothesized checkpoint inhibitor‐ induced T cell‐mediated conditions which affect the skin, gut, and liver.^11^, ^12^ Indeed, the concept of an autoantibody‐mediated condition is supported by the recent discovery of increased plasmablasts,^13^ and a few organ‐specific autoantibodies, post‐checkpoint inhibitors.^14^

In addition to hypophysitis, neurological side effects of the checkpoint inhibitors include very few or single cases of aseptic meningitis, GBS, and myositis.^5^, ^15^ More recently, 0.1% of patients administered nivolumab were reported to develop MG. Furthermore, multiple sclerosis after CTLA‐4 blockade by ipilimumab has been reported in one case where the clonally expanded T cell receptor sequences shared between the CSF and melanoma suggest preformed T cell memory led to the CNS disease.^16^ Similar close clonal relationships of T cells are recognized between tumor and cardiac tissue in post‐checkpoint inhibitor myocarditis,^12^ supporting the notion that many irAEs are T cell‐mediated.

Overall, the consistent 4‐week lag observed by us and two cases with autoimmune encephalitis after a similar interval from immune checkpoint inhibitor administration,^17^ suggest insufficient time for generation of a de novo immune response to neurological antigens. More likely, the drug‐induced disinhibition of circulating T cells led to the activation of preformed B cell reactivities to neural and neuromuscular proteins. This observation implies that autoantigen‐specific T and B cells were circulating in an anergic or quiescent state, contained by Tregs: indeed, flow cytometry from patient 1 suggests the suppressive population were within the CD4^+^CD25^+^CD62L^+^ Tregs bound by the monoclonal antibody pembrolizumab. Interestingly, the presence of preexisting neural antigen‐specific cells in those with or without disease may be consistent with the frequent detection of neurological autoantibodies, particularly of the IgM subclass, in healthy control subjects.^18^ Similarly, screening of patients without neurological symptoms who have tumors and paraneoplastic autoantibodies, for example, GABA_B_‐receptor antibodies and small cell lung carcinoma,^19^ may identify those with a potentially increased rate of irAEs. As an alternative to a T cell‐directed mechanism of action, as PD‐1 and CTLA‐4 are also expressed on B cells and some myeloid cells, and PD‐L1 on neurons and tumor cells, the checkpoint inhibitors may have additional direct actions via other cell types.^2^ These mechanisms should be investigated in future studies.

In summary, antibody‐mediated neurological complications require increased clinical vigilance given the escalating use of immune checkpoint inhibitors. Further study of these patients may highlight immunological mechanisms operating in all antibody‐mediated diseases, and have implications for the detection of anergic autoreactive T and B cells in healthy controls.

## Author Contributions

RW, AD, JH, DM, JC, DB, WK, PM, SM, SR, GC, and NS were involved in data collection, drafting, and analysis revising manuscript for intellectual content; SJ and SRI were involved in study conceptualization, data collection, drafting, analysis, and revising the manuscript for intellectual content.

## Conflicts of Interest

GC has received honoraria from Merck and BMS for advisory work and trial funding; SRI receives royalties as a coapplicant on a patent describing VGKC‐complex antibodies, including LGI1, CASPR2, and contactin‐2, licensed to Euroimmun Ltd.

## Supporting information


**Table S1**. Unremarkable investigations in patients. *full blood count, electrolytes, liver function tests, bone profile; **erythrocyte sedimentation rate, C‐reactive protein, antineutrophil cytoplasmic antibodies, antinuclear antibodies; ***Hu, Yo, Ri, CRMP5, glutamic acid decarboxylase, LGI1, CASPR2, MAG, ganglioside screen; ****CMV, EBV, Hepatitis A/B/C, HIV, Borrelia IgMs were negative.
