# Supplementary material for: Seronegative antibody‐mediated neurology after immune checkpoint inhibitors
Source: Ann Clin Transl Neurol. 2018 Mar 25;5(5):640–5. doi: 10.1002/acn3.547 (PMC5945956; doi:10.1002/acn3.547)
Supplement: Supplementary file 1 — Table S1. Unremarkable investigations in patients. *full blood count, electrolytes, liver function tests, bone profile; **erythrocyte sedimentation rate, C‐reactive protein, antineutrophil cytoplasmic antibodies, antinuclear antibodies; ***Hu, Yo, Ri, CRMP5, glutamic acid decarboxylase, LGI1, CASPR2, MAG, ganglioside screen; ****CMV, EBV, Hepatitis A/B/C, HIV, Borrelia IgMs were negative. [file ACN3-5-640-s001.docx]

|  | Patient 1 | Patient 2 | Patient 3 | Patient 4 |
| --- | --- | --- | --- | --- |
| Basic blood work* | Unremarkable | Unremarkable | Unremarkable | Unremarkable |
| Vasculitic screen** | Unremarkable | Unremarkable | Unremarkable | Unremarkable |
| Neuronal autoantibodies*** | Unremarkable | Unremarkable | Unremarkable | Unremarkable |
| CSF | Lymphocytosis only | Not performed | Not performed | High protein only |
| Infectious screen**** | Unremarkable | Unremarkable | Unremarkable | Unremarkable |
| MRI brain | Figure 1 | Normal | Three metastases (temporal and frontal lobe) | Hypophysitis; spine MRI normal |

**Supplementary Table 1**. Unremarkable investigations in patients. *full blood count, electrolytes, liver function tests, bone profile; **erythrocyte sedimentation rate, C-reactive protein, anti-neutrophil cytoplasmic antibodies, anti-nuclear antibodies; ***Hu, Yo, Ri, CRMP5, glutamic acid decarboxylase, LGI1, CASPR2, MAG, ganglioside screen; ****CMV, EBV, Hepatitis A/B/C, HIV, Borrelia IgMs were negative.
